# Evaluating the Dual-Target Aptima HIV-1 Quant Dx Assay: Comparison between Viral Loads Measured with *pol* and *LTR* Targets in the Same Samples

**DOI:** 10.1128/spectrum.01361-22

**Published:** 2022-09-06

**Authors:** Giuseppe Sberna, Silvia Sarti, Stefania Cicalini, Andrea Antinori, Anna Rosa Garbuglia, Alessandra Amendola

**Affiliations:** a Laboratory of Virology, National Institute for Infectious Diseases “Lazzaro Spallanzani” IRCCS, Rome, Italy; b HIV/AIDS Department, National Institute for Infectious Diseases “Lazzaro Spallanzani” IRCCS, Rome, Italy; University of Cincinnati

**Keywords:** HIV-1 RNA monitoring, viral load, Aptima HIV-1 Quant Dx assay, dual-target assay, antiretroviral therapy, LTR

## Abstract

For effective management of HIV-1 patients, accurate measurement of HIV-1-RNA viral load (VL) is fundamental. The latest generation molecular assays for monitoring VL perform simultaneous detection of two regions of the viral genome, but without specifying the target used for VL quantitation. By using the “open” software (research use only [RUO]) of Aptima HIV-1 Quant Dx Assay (Aptima) which provides both results obtained with the *pol* and *LTR* targets, we were able to compare *n* = 500 plasma samples results from chronically HIV-1-infected patients under antiretroviral therapy (ART). Correlation and concordance were analyzed. By stratifying VL into two groups (<30 and ≥30 copies/mL HIV-1-RNA) according to *pol-*based results and matching them with their respective *LTR* values, concordance was substantial (κ = 0.635; 95%CI = 0.569 to 0.700) as expected. Considering the specimens (*n* = 224) with VL exactly quantified (i.e., ≥30 copies/mL) with both targets, an optimal correlation subsisted (*r* = 0.8882; *P* < 0.0001) and Bland-Altman plot showed no significant mean difference between them. However, by stratifying all these data in three ranges (30 to 200, 201 to 1,000, and >1,000 copies/mL) according to *pol-*based results, concordance analysis showed fair agreement (κ = 0.344; 95%CI = 0.257 to 0.432). Indeed, after excluding mutually concordant VL values in each range (*n* = 134), the remaining discordant samples (*n* = 90; 40.1%) showed significant (*P* < 0.05) difference between VL measured with the two targets. With the Aptima “open” software, samples with *pol*-based VL <1,000 copies (cp)/mL HIV-1-RNA, the corresponding *LTR* values were on average 0.5 log_10_ cp/mL higher. Further studies on these discrepancies and the nature of viral RNA elements detected only with the *LTR* despite efficient ART are in progress.

**IMPORTANCE** The last generation dual-target platforms for quantification of HIV-1 RNA return a single value of viral load (VL) derived from a combined reading of two HIV-1 genome targets. By using a modified version of Aptima software, providing both the VL results obtained from *pol* and *LTR* amplification separately, we observed discordant VL results in some samples from HIV-1-infected patients on antiretroviral therapy. In particular, some samples with *pol*-based quantified <1,000 copies/mL VL showed the *LTR-*based value on average 0.5 log_10_ copies/mL higher, and other samples, always by treated patients, showed VL exclusively quantified with *LTR* target while the corresponding *pol*-based VL results were completely undetected. Standard software of double-target based diagnostic systems does not allow recognizing discrepant VL values in these particular, but not rare, clinical specimens. This issue could have implications for clinical management by leading physicians to consider changing antiretroviral regimen based on presumed failure of antiretroviral therapy.

## INTRODUCTION

For the correct and the effective management of early and chronic HIV-1-infected patients, an accurate measurement of HIV-1 RNA viral load (VL) is fundamental before starting of antiretroviral therapy (ART) and in monitoring antiretroviral treatment efficacy (1).

Most available HIV-1 RNA tests are based on real-time PCR and are designed for the detection of a single target region of HIV-1 genome (i.e., *pol*, *gag*, *LTR*). However, the latest generation molecular assays have been set on the simultaneous detection of two regions of viral genome to ensure the recognition of the virus even in the presence of mutations. One of these diagnostic assays is the Aptima HIV-1 Quant Dx Assay (Aptima; Hologic, Inc., San Diego, CA), a totally automated assay based on transcription mediated amplification that utilizes multiple HIV-1-specific primers to capture two regions of the HIV-1 genome. The detection of viral genome is performed in a multiplex reaction, in which both *pol* and *LTR* regions of HIV-1 are simultaneously and independently amplified (2) with high efficiency in detecting and quantifying VL (3–10). A feature of Aptima software is, although two VL values are produced at the end of testing, one related to *pol* and the other related to *LTR* amplification, the result returned of viremia is the one related to the target *pol* by default, independently of *LTR*-based values of HIV-1 RNA. Whether *pol* signal is not detected (ND) and *LTR* target is detected, the latter result is reported as the final VL value. However, the information about the target used for quantitation is available at the level of individual sample report, and not in the overall run report.

In a previous study (7), we reported that 6% of plasma samples from chronically HIV-1-infected patients under effective ART show absence of *pol* signal with viremia values calculated exclusively on the basis of *LTR* amplification. Despite VL detected only by the *LTR* target, these patients were in excellent and stable clinical condition for which, based on numerous clinical and laboratory parameters, the ART was considered effective and not modified, even with *LTR* VL >1,000 copies/mL (unpublished data).

In this study, we compared the VL paired results obtained by *pol* and *LTR* amplification of HIV-1 RNA in plasma samples of ART-treated patients under routine monitoring of viremia. We analyzed these VL paired results in each sample, in terms of correlation and concordance, to evaluate the level of agreement between them. This analysis was performed using an “open” version of Aptima software (RUO), provided by the test manufacturer (Hologic, Inc.) and configured to show both VL results for each individual target amplified on HIV-1 RNA.

## RESULTS

### Comparison between the VL values measured with *pol* and *LTR* targets in the same samples.

An intra-assay comparative evaluation of VL values measured by *pol* and *LTR* targets amplification in the same specimens was conducted because we noticed that 24% of samples showed a significant difference (>0.3 log_10_ copies (cp)/mL HIV-1 RNA) between VL detected with *pol* and *LTR* targets. The analysis was carried out by using VL values referred to *n* = 500 plasma from ART-treated HIV-1-infected patients under routine viremia monitoring. The VL data were collected consecutively, from routine diagnostic activity, according to the *pol* amplification values: i.e., *n* = 100 results with VL ND; *n* = 100 results with VL detected <30 cp/mL HIV-1 RNA; *n* = 100 VL resulted in the range 30 to 200 cp/mL; *n* = 100 with VL 201 to 1,000 cp/mL and *n* = 100 VL with >1,000 cp/mL HIV-1 RNA. These ranges of *pol*-based viremia were established after considering the international guidelines for the management of HIV-1 infection (11, 12), which indicate the virological failure as a confirmed viral load >200 or >1,000 copies/mL of HIV-1 RNA.

First, for the comparison analysis, all the collected samples were divided into two groups according to following ranges of *pol* VL (ND plus <30 and ≥30 cp/mL HIV-1 RNA) and matched with their respective *LTR*-based values. The concordance analysis (Table 1) showed a “substantial agreement” for HIV-1 RNA detection between the two targets (κ = 0.635; 95% CI = 0.569 to 0.700). Among samples with *pol* VL ND, five showed HIV-1 RNA detected <30 cp/mL with *LTR* target and one had viremia value of 1,284 cp/mL because of the *LTR* amplification.

**TABLE 1:** Distribution of samples (*n* = 500) according to their VL values measured with *pol* and *LTR* amplification by using Aptima “open” software

No. of samples^*a*^	*Pol-*based VL values		
	<30	≥30	Total
*LTR-*based VL values			
<30	184	76	260
≥30	16	224	240
Total	200	300	500

a Samples were stratified according to their VL values in two ranges (ND plus <30 and >30 cp/mL HIV-1 RNA).

### Correlation between paired VL values measured with *pol* and *LTR* targets in the same samples with viremia exactly quantified with both the targets.

**FIG 1:**
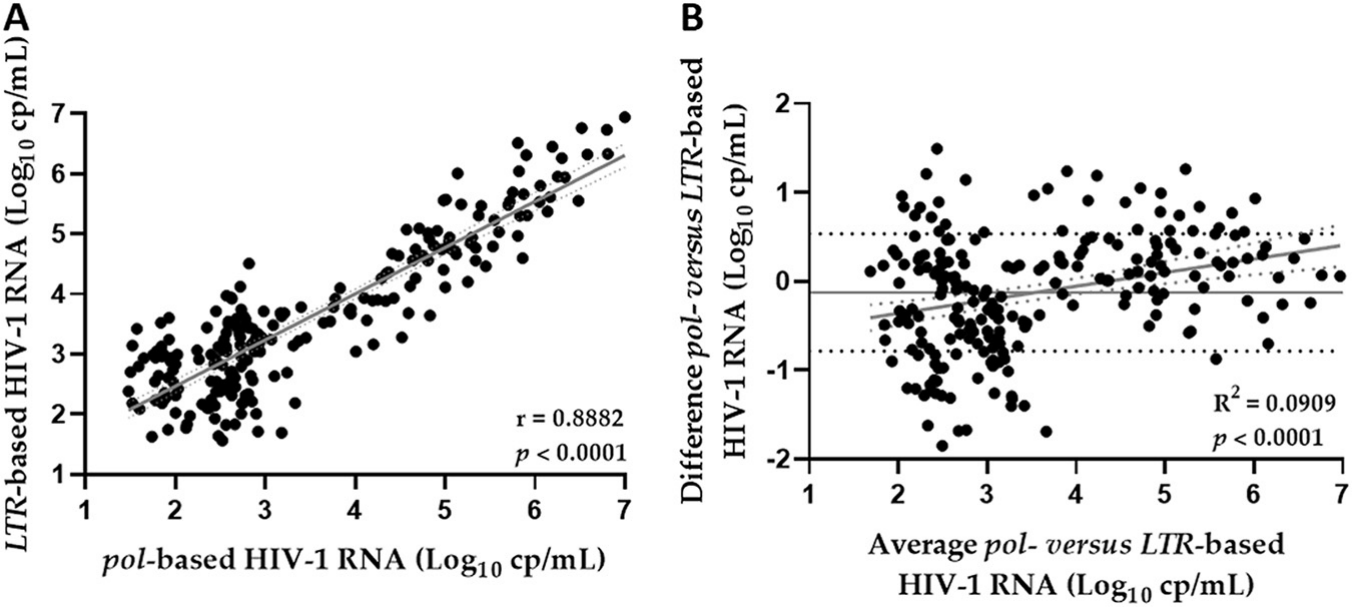
Correlation and Bland-Altman plot of paired VL values measured with *pol* and *LTR* targets in the same samples with viremia exactly quantified by both the targets. (A) Correlation between *pol* and *LTR* VL values measured in *n* = 224 samples. (B) Bland-Altman plot shows average values of *pol* and *LTR* VL paired values measured in each sample *versus* the difference between them in the same samples.

The correlation between *pol* and *LTR* viremia has been carried out by including all samples (*n* = 224) with the HIV-1 RNA VL exactly quantified with both the targets (i.e., >30 cp/mL). As shown in Fig. 1A, correlation (*r* = 0.8882; *P* < 0.0001) between values was high in linear regression analysis. In Fig. 1B, Bland-Altman plot analysis showed an average difference between the VL measured with *pol* and *LTR* amplification in the same sample of 0.124 log_10_ cp/mL (±SD = ±0.659 log_10_ cp/mL; 95% CI = −1,417 to 1,168). Interestingly, in the range of *pol*-based 4 to 6 log_10_ cp/mL HIV-1 RNA, including *n* = 64 (27.68%) samples out of 224, the viremia quantified with *LTR* target tended to be lower (mean ± SD = 4.99 ± 0.54 *pol*-based *versus* 4.73 ± 0.73 *LTR*-based log_10_ cp/mL; *P = *0.0253). On the contrary, in the range of *pol*-based 2 to 4 log_10_ cp/mL (mean ± SD = 2.74 ± 0.42 log_10_ cp/mL) including *n* = 115 (51.34%) samples, the *LTR*-based VL (mean ± SD = 2.93 ± 0.66 log_10_ cp/mL) was 0.22 log_10_ cp/mL higher (*P = *0.0170).

### Comparison of VL values exactly quantified with both the targets *pol* and *LTR* ≥30 cp/mL.

By stratifying the VL data measured by *pol* in the ranges 30 to 200, 201 to 1,000, and >1,000 cp/mL HIV-1 RNA, concordance analysis with corresponding values measured by *LTR* target in the same samples showed “fair agreement” (κ = 0.344; 95% CI = 0.257 to 0.432) (Table 2).

**TABLE 2:** Distribution of the samples (*n* = 224) with VL exactly quantified with both *LTR* and *pol* targets

No. of samples^*a*^	*Pol-*based VL values			
	30 to 200	201 to 1,000	>1,000	Total
*LTR-*based VL values				
30 to 200	13	20	2	35
201 to 1,000	20	26	2	48
>1,000	8	38	95	141
Total	41	84	99	224

a Samples were stratified according to their VL values in three ranges of HIV-1 RNA: 30 to 200, 201 to 1,000, and >1,000 cp/mL.

**FIG 2:**
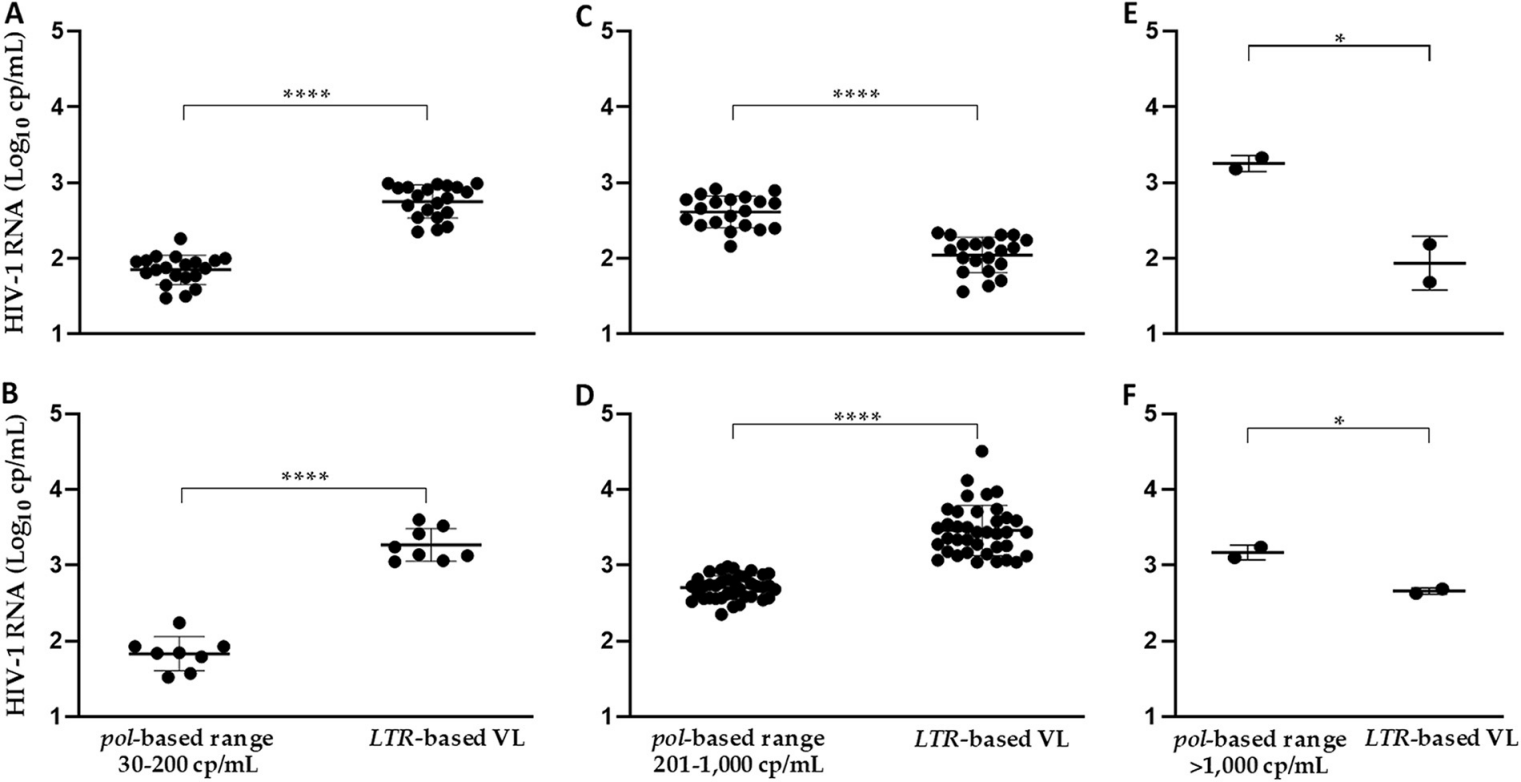
Graphic representation of *pol* and *LTR* VL measured in discordant samples stratified in three ranges of *pol*-based VL. *Pol*-based VL data were stratified within three ranges of VL (30 to 200, 201 to 1,000, and >1,000 cp/mL HIV-1 RNA) and, for each *pol*-based range, the corresponding VL based on *LTR* amplification were shown in two graphs separately to display their range of VL. (A) and (B) Discordant samples with *pol*-based VL included in the range 30 to 200 cp/mL HIV-1 RNA. (A) Samples with their corresponding *LTR*-based VL included in the ranges 201 to 1,000 cp/mL. (B) Corresponding *LTR*-based VL >1,000 cp/mL. (C) and (D) Discordant samples with *pol*-based VL included in the range 201 to 1,000 cp/mL HIV-1 RNA. (C) Samples with their corresponding *LTR*-based VL in the range 30 to 201 cp/mL. (D) Those with their corresponding *LTR*-based VL >1,000 cp/mL. (E) and (F) Samples with *pol*-based VL >1,000 cp/mL HIV-1 RNA. (E) Samples with their corresponding *LTR*-based VL in the range 30 to 201 cp/mL. (F) Samples with their corresponding *LTR*-based VL in the range 201 to 1,000 cp/mL. Bars in the graphs show mean and standard deviation; asterisks indicate statistically significant difference, as determined by Student's *t* test (****, *P* < 0.0001; *, *P* < 0.05).

By excluding all mutually concordant VL values of each range (*n* = 134; 59.82%), the remaining samples showed discrepancy between VL measured by *pol* amplification and that measured by *LTR* amplification (mean ± SD = 2.44 ± 0.45 *pol*-based *versus* 2.92 ± 0.64 *LTR*-based log_10_ cp/mL; *P* < 0.0001) within their own group. The discrepancy affects *n* = 28 of 41 (68.3%) and *n* = 58 of 84 (69.0%) samples in the two ranges of 30 to 200 cp/mL and 201 to 1,000 cp/mL of *pol*-based VL, respectively, and only *n* = 4 (4.0%) samples in the range of *pol*-based >1,000 cp/mL HIV-1 RNA. Interestingly, in the *pol*-based range of VL 201 to 1,000 cp/mL (mean ± SD = 2.64 ± 0.17), the largest number of discordant samples (*n* = 38 out of 84; 45.2%) showed *LTR*-based viremia >1,000 cp/mL (mean ± SD = 3.46 ± 0.33 log_10_ cp/mL). In the other group, with lower range of *pol*-based 30 to 200 cp/mL HIV-1 RNA (mean ± SD = 1.86 ± 0.20), 48.8% of samples (*n* = 20 of 41) showed *LTR*-based VL extended between 201 and 1,000 cp/mL (mean ± SD = 2.75 ± 0.21 log_10_ cp/mL) and 19.5% of samples (*n* = 8 out of 41) showed *LTR*-based viremia >1,000 cp/mL (mean ± SD = 3.27 ± 0.21 log_10_ cp/mL). Therefore, in both the two low ranges groups of *pol*-based VL, most of discordant samples (*n* = 28 and *n* = 58) showed higher mean *LTR* titers respect to the corresponding mean *pol* values (mean ± SD = 2.40 ± 0.43 *pol*-based *versus* 2.94 ± 0.63 *LTR*-based log_10_ cp/mL; *P* < 0.0001) (Fig. 2). In the range of 30 to 200 cp/mL of *pol*-based VL, the mean difference between VL measured with *pol* and *LTR* was −1.1 log_10_ cp/mL HIV-1 RNA (min-max of difference values of −1.9 and −0.4 log_10_ cp/mL HIV-1 RNA, respectively). In the range of 201 to 1,000 cp/mL, the mean difference was −0.3 log_10_ cp/mL HIV-1 RNA (min-max of difference values of −1.7 and 1.2 log_10_ cp/mL HIV-1 RNA, respectively).

Furthermore, considering the discordant samples with *pol* values >1,000 cp/mL, the mean difference between VL measured with *pol* and *LTR* was −0.9 log_10_ cp/mL HIV-1 RNA (min-max of difference values of 0.5 and 1.5 log_10_ cp/mL HIV-1 RNA, respectively).

## DISCUSSION

Quantification of HIV-1 RNA is an essential parameter in clinical management of HIV-1-infected patients and understanding the performance of molecular HIV-1 assays is necessary for correct interpretation of results (1).

The latest generation analytical platforms are based on dual-target detection, whereby the VL value derives from the simultaneous reading of the amplification products of two specific regions present on HIV-1 RNA. Most of these systems return values of VL in an unspecified manner and they do not declare separately the VL measured with each target. The CE-IVD version of Aptima software also follows this reporting mode, but, upon request by the user, it is possible to receive a modified version of the software (RUO) from the manufacturer, providing both the VL results derived from the amplification of each of the two targets.

By using the “open” version of the Aptima software, we compared the VL data obtained by amplification of *pol* and *LTR* targets in the same samples. We reported that, considering all the specimens with VL exactly quantified (i.e., ≥30 cp/mL HIV-1 RNA) with both targets, an optimal correlation subsists, as expected, and indeed Bland-Altman plot showed not significant mean difference between them.

However, by analyzing the results in more detail, in a set of samples we observed the paired values of VL did not always overlap exactly between them. In fact, in the samples with *pol*-based viremia of 4 to 6 log_10_ cp/mL, the corresponding *LTR* VL were significantly lower, while, in the other range with *pol*-based VL of 2 to 4 log_10_ cp/mL, the corresponding *LTR* values were significantly higher.

In particular, discordant specimens with *pol* quantified VL below 1,000 cp/mL showed corresponding *LTR* values that were on average 0.5 log_10_ cp/mL significantly higher. On the contrary, considering the discordant samples with *pol* values >1,000 cp/mL, the LTR values were on average 0.9 log_10_ cp/mL significantly lower than the *pol*-based values.

Differently from Aptima equipped with “open” software, currently available dual-target diagnostic systems do not specify the HIV-1 RNA values measured with each of the two targets because they are based on the use of a single fluorescent probe, common to both amplified HIV-1 regions, so they return single viremia results. These kind of results may have serious implications in clinical management of infected patients. In fact, in the presence of VL values >200 or >1,000 cp/mL HIV-1 RNA, physicians may consider changing of therapeutic regimen based on the hypothesis of a presumed failure of antiretroviral therapy, according to indications of main international guidelines (11, 12). Conversely, having a system that indicates when VL is detected with only one of the two HIV-1 targets may allow the clinician to consider the partial VL result and decide to repeat the HIV-1 RNA quantification sometime afterwards, or even with another diagnostic system, as well as to carry out further investigations on the clinical situation before changing the therapeutic regimen. Unfortunately, current guidelines for the management of HIV-infected patients do not take into consideration the issues correlated with double target detection; in particular, the guidelines do not mention situations in which a test can return quantitative VL based on amplification of a single target (while the other is absent) without a warning signal when the two amplification curves are discordant.

Here, and in a previous work (7), we showed some HIV-1-infected patients (6%) have VL exactly quantified deriving exclusively from the amplification of the *LTR* target, while the *pol* target is completely undetected. This situation always occurs in patients under ART, regardless of the type of therapeutic regimen (7). Furthermore, because clinical conditions and laboratory parameters of these patients remained, and continue to be stable and compatible with a picture suggestive of effective antiretroviral therapy (7; unpublished data), ART has, to date, never been changed in these patients. However, it is important to note that if for some reason a patient could not be tested with this “open” Aptima configuration, finding a quantified VL with different assay would not allow the clinician to understand the target used for the quantification of VL and could raise unfounded doubts about the efficacy of the therapeutic regimen and the patient's adherence to therapy. Therefore, in our opinion, the target amplified for measurement of VL should be carefully considered during clinical evaluation of laboratory data in order to avoid any inappropriate changes in the therapeutic regimen when VL are detected only on the basis of *LTR* target. This highlights the need to have diagnostic systems for monitoring the HIV viral load that make it possible to know both viremia values obtained with the two targets separately, as in the case of the “open” version of the Aptima software.

Furthermore, here we have described, in a subset of samples with HIV-1 RNA VL <1000 cp/mL with target *pol*, a gradient of *LTR*-based viremia, thus reinforcing the potential clinical utility of having access to both, separate VL results, from *pol* and *LTR*, especially for those samples detected only through the *LTR* target.

We do not know yet the nature of these *LTR*-only-based viral RNA products. It could be they reflect a therapeutic regimen not completely effective, or it could be the consequence of other causes, such as presence of HIV-1 *reservoir* with proviral DNA partially transcribed through effective ART; *reservoir* with proviral DNA inserted in highly expressed sites of the host cells favoring the initiation of transcription, which then stops producing short RNA containing only *LTR* region or *LTR* and other part of HIV-1 RNA without standard *pol* sequence; or *reservoirs* containing circles of DNA producing incomplete *LTR* transcripts without the *pol* region (13–15).

Recently, in HIV-1 seronegative cancer patients under chimeric antigen receptor (CAR) T-cell immunotherapy, it has been described false HIV-1 nucleic acid amplification with a double-targets assay different from Aptima (16, 17). This was attributed to the detection of LTR elements present in lentiviral vectors used in this type of immunotherapy (16, 17). In our experience, described here, no patient has been or was subjected to CAR T-cell immunotherapy; therefore, we can exclude that any LTR signal can be traced back to the presence of lentiviral vectors.

Therefore, studies aimed to elucidate the nature, the origin, and the function of these HIV-1 RNA transcripts, recognized only by the *LTR* signal in plasma samples of chronically HIV-1-infected patients under ART, are in progress to understand pathogenic and therapeutic aspects associated with the detection of these elements and to further find out if they could have particular implication on clinical management.

## MATERIALS AND METHODS

### Clinical samples.

Plasma samples were collected from chronically HIV-1-infected patients under ART, attending the outpatient care facility at National Institute for Infectious Diseases “Lazzaro Spallanzani” IRCCS in Rome for routine monitoring of HIV-1 from December 3, 2018 to September 8, 2019. Plasma were obtained from whole blood collected in EDTA-containing tubes by centrifugation (1,100 g for 20 min) directly after arrival at the laboratory and tested same day or within the subsequent day with Aptima.

### Aptima HIV-1 Quant Dx assay.

Aptima was used on the fully automated Panther system according to the manufacturers’ instructions. The assay requires a sample volume of 0.7 mL and reports quantitative HIV-1 results in a range of 30 to 10,000,000 copies/mL (cp/mL). Samples with HIV-1 RNA detected below the low limit of quantification (LLoQ) (30 cp/mL) and those with HIV-1 RNA ND (i.e., with HIV-1 RNA below the low limit of detection [LLoD], 12 cp/mL) were indicated as <30 cp/mL. The 7.2.1.37 “open” version of the Aptima software showing *pol*- and *LTR*-based viremia values separately was provided by the assay manufacturer per our request and routinely used.

### Statistical analysis.

Data management and analyses were performed using GraphPad Prism version 8.00 (GraphPad Software, La Jolla, CA, USA). The evaluation of the qualitative concordance between results was performed using the weighted Cohen's kappa statistics and its 95% CI. Correlation analyses were carried out using a linear regression analysis. Student's *t* test (Welch's *t* test) was used for difference evaluation. For statistical calculations, an arbitrary value of 15 cp/mL was assigned to samples detected with VL <30 cp/mL.

### Institutional review board statement.

The study was conducted in accordance with the Declaration of Helsinki and with the protocol code no. 70, approved on December 17, 2018 by the Institutional Review Board of the National Institute for Infectious Diseases L. Spallanzani, IRCCS, according to which the study protocol here described did not provide for the signing of an informed consent by the patients because no further samples were taken other than those performed for diagnostic purposes. Data of biological samples collected for diagnostic purposes were used only after their complete anonymization and the results of tests had no impact on the clinical management of patients. Furthermore, the analysis of genetic data were not provided.

## References

[1] European AIDS Clinical Society (EACS) Guidelines. 2021. https://www.eacsociety.org/guidelines/eacs-guidelines/.

[2] Aptima HIV-1 Quant Dx assay package insert. https://www.hologic.com/sites/default/files/package-insert/AW-11853-001_003_01.pdf.

[3] Amendola A, Pisciotta M, Aleo L, Ferraioli V, Angeletti C, Capobianchi MR. 2016. Evaluation of the Aptima() HIV-1 Quant Dx assay for HIV-1 RNA viral load detection and quantitation in plasma of HIV-1-infected individuals: a comparison with Abbott RealTime HIV-1 assay. J Med Virol 88:1535–1544. doi:10.1002/jmv.24493. 26864171 PMC6585778

[4] Schønning K, Johansen K, Landt B, Benfield T, Westh H. 2017. Comparison of the Hologic Aptima HIV-1 Quant Dx Assay to the Roche COBAS Ampliprep/COBAS TaqMan HIV-1 Test v2.0 for the quantification of HIV-1 RNA in plasma samples. J Clin Virol 92:14–19. doi:10.1016/j.jcv.2017.05.006. 28505569

[5] Sauné K, Raymond S, Boineau J, Pasquier C, Izopet J. 2016. Detection and quantification of HIV-1 RNA with a fully automated transcription-mediated-amplification assay. J Clin Virol 84:70–73. doi:10.1016/j.jcv.2016.09.002. 27728849

[6] Hatzakis A, Papachristou H, Nair SJ, Fortunko J, Foote T, Kim H, Peling TL, Worlock AJ. 2016. Analytical characteristics and comparative evaluation of Aptima HIV-1 Quant Dx assay with Ampliprep/COBAS TaqMan HIV-1 test v2.0. Virol J 13:176. doi:10.1186/s12985-016-0627-y. 27769309 PMC5073876

[7] Amendola A, Sberna G, Forbici F, Abbate I, Lorenzini P, Pinnetti C, Antinori A, Capobianchi MR. 2020. The dual-target approach in viral HIV-1 viremia testing: an added value to virological monitoring? PLoS One 15:e0228192. doi:10.1371/journal.pone.0228192. 32023284 PMC7001951

[8] Sam SS, Kurpewski JR, Cu-Uvin S, Caliendo AM. 2016. Evaluation of performance characteristics of the Aptima HIV-1 Quant Dx assay for detection and quantitation of human immunodeficiency virus type 1 in plasma and cervicovaginal lavage samples. J Clin Microbiol 54:1036–1041. doi:10.1128/JCM.03289-15. 26842702 PMC4809915

[9] Aretzweiler G, Leuchter S, García-Álvarez M, Simon C, Marins E, Paxinos E, Canchola J, Delgado R, Frontzek A. 2019. Analytical performance of four molecular platforms used for HIV-1, HBV and HCV viral load determinations. Expert Rev Mol Diagn 19:941–949. 31159598 10.1080/14737159.2019.1624162

[10] Yek C, Massanella M, Peling T, Lednovich K, Nair SV, Worlock A, Vargas M, Gianella S, Ellis RJ, Strain MC, Busch MP, Nugent CT, Richman DD. 2017. Evaluation of the Aptima HIV-1 Quant Dx assay for HIV-1 RNA quantitation in different biological specimen types. J Clin Microbiol 55:2544–2553. doi:10.1128/JCM.00425-17. 28592548 PMC5527433

[11] World Health Organization. 2021. Consolidated guidelines on HIV prevention, testing, treatment, service delivery andmonitoring: recommendations for a public health approach. https://www.who.int/publications/i/item/9789240031593. 34370423

[12] Panel on Antiretroviral Guidelines for Adults and Adolescents. 2021. Guidelines for the Use of Antiretroviral Agents in Adults and Adolescents with HIV. Department of Health and Human Services. https://clinicalinfo.hiv.gov/sites/default/files/guidelines/archive/AdultandAdolescentGL_2021_08_16.pdf.

[13] Ho YC, Shan L, Hosmane NN, Wang J, Laskey SB, Rosenbloom DI, Lai J, Blankson JN, Siliciano JD, Siliciano RF. 2013. Replication-competent noninduced proviruses in the latent reservoir increase barrier to HIV-1 cure. Cell 155:540–551. doi:10.1016/j.cell.2013.09.020. 24243014 PMC3896327

[14] Bruner KM, Murray AJ, Pollack RA, Soliman MG, Laskey SB, Capoferri AA, Lai J, Strain MC, Lada SM, Hoh R, Ho YC, Richman DD, Deeks SG, Siliciano JD, Siliciano RF. 2016. Defective proviruses rapidly accumulate during acute HIV-1 infection. Nat Med 22:1043–1049. doi:10.1038/nm.4156. 27500724 PMC5014606

[15] Imamichi H, Dewar RL, Adelsberger JW, Rehm CA, O’Doherty U, Paxinos EE, Fauci AS, Lane HC. 2016. Defective HIV-1 proviruses produce novel protein-coding RNA species in HIV-infected patients on combination antiretroviral therapy. Proc Natl Acad Sci USA 113:8783–8788. doi:10.1073/pnas.1609057113. 27432972 PMC4978246

[16] Ariza-Heredia EJ, Granwehr BP, Viola GM, Bhatti M, Kelley JM, Kochenderfer J, Hosing C. 2017. False-positive HIV nucleic acid amplification testing during CAR T-cell therapy. Diagn Microbiol Infect Dis 88:305–307. doi:10.1016/j.diagmicrobio.2017.05.016. 28610774 PMC7891749

[17] Hauser JR, Hong H, Babady NE, Papanicolaou GA, Tang YW. 2019. False-positive results for human immunodeficiency virus type 1 nucleic acid amplification testing in chimeric antigen receptor T cell therapy. J Clin Microbiol 58:e01420-19. doi:10.1128/JCM.01420-19. 31694968 PMC6935905

